# Target categorization with primes that vary in both congruency and sense modality

**DOI:** 10.3389/fpsyg.2015.00020

**Published:** 2015-01-23

**Authors:** Kathryn Weatherford, Michael Mills, Anne M. Porter, Paula Goolkasian

**Affiliations:** Department of Psychology, University of North Carolina at CharlotteCharlotte, NC, USA

**Keywords:** cross-modal, conceptual priming, categorization task, perception, semantic processing

## Abstract

In two experiments we examined conceptual priming within and across sense modalities by varying the modality (picture and environmental sounds) and the category congruency of prime-target pairs. Both experiments used a repetition priming paradigm, but Experiment 1 studied priming effects with a task that required a superordinate categorization response (man-made or natural), while Experiment 2 used a lower level category response (musical instruments or animal): one that was more closely associated with the basic level of the semantic network. Results from Experiment 1 showed a strong effect of target modality and two distinct patterns of conceptual priming effects with picture and environmental sound targets. However, no priming advantage was found when congruent and incongruent primes were compared. Results from Experiment 2, found congruency effects that were specific to environmental sound targets when preceded by picture primes. The findings provide support for the intermodal event file and multisensory framework, and suggest that auditory and visual features about a single item in a conceptual category may be more tightly connected than two different items from the same category.

## INTRODUCTION

In previous research with pictures and environmental sounds (Kim et al., 2014), we examined conceptual priming within and across modalities and found that target categorization was facilitated by the advanced presentation of conceptually related exemplars, but there were differences in the effectiveness of visual and auditory primes. The present study follows up this research by varying the congruence between the prime-target pairs to explore cross-modal priming in a more comprehensive manner.

In conceptual priming, target facilitation is based on processing the semantic relationship between the prime and the target, and it differs from perceptual priming, which is based on the advanced presentation of some aspect of the physical stimulus. The primary concern in our research is whether categorization of pictures and environmental sounds would be similarly facilitated by the advanced presentation of stimuli that varied in modality and were derived from same or different items within the same semantic category as the targets: would the sound of a dog barking or a cat meowing facilitate categorization of a picture of a dog in the same way as another picture of a dog or a cat? Of secondary interest is the difference in processing targets presented in either visual or auditory formats.

Perception is naturally accustomed to multisensory experiences that include sensory interactions and a mechanism to bind them into a coherent perceptual representation. A classic example of a binding mechanism is an object file, introduced by Kahneman et al. (1992) and updated by Hommel (1998, 2004, 2005) with the event file. They explained how features are bound to objects and enriched by object-related knowledge from long-term memory. Work by Zmigrod et al. (2009) suggests that auditory and visual features can be integrated and bound with each other and with their associated response. Their data show that feature integration operates across perceptual domains. Sensory integration occurs with an event file, which is a network of bindings that link codes of the salient features of a perceptual event. Repeated encounters with an event file produce retrieval of the event file in such a way that it may facilitate performance, if the same stimulus event is experienced; or it may interfere with the creation of new event files that share some but not all of the same features.

Chen and Spence’s (2011) work also establishes a mechanism by which visual and auditory information can access semantic information. They extended Glaser and Glaser’s (1989) picture/word model to sounds and suggested that naturalistic sounds and pictures access semantic representations automatically while spoken and printed words access a corresponding lexical representation. Support for Chen and Spence’s (2011) multisensory framework is provided by a series of their studies that show an enhancement of picture detection when associated sounds are presented at least 349 ms prior to the presentation of a picture target.

If sensory information is integrated and bound with each other into an event file or stored in a multisensory framework, we would expect target categorization to be influenced whether it was preceded by a prime from the same or a different sense modality. The nature of the influence may vary, however, depending upon variation in processing stimulus material from picture and environmental sound formats. It is well known that picture processing is immediate with global information followed by local while sounds unfold in a sequential manner such that feature acquisition occurs across time.

Not all models of knowledge representation, however, are compatible with a multisensory framework. Some (Pylyshyn, 1999; Riesenhuber and Poggio, 2000) suggest that auditory and visual processing are modular and accessible to each other only through a symbolic or amodal representation. So, essentially audio-visual features of conceptual objects would not be integrated or retained, only processed with their meaning abstracted. A priming study that compares modality and semantic congruity effects would have some implications for the debate regarding how multisensory experiences are related. In repetition priming, the prime is presented in advance of the target and response times (RTs) to the target would reflect any influence that the prime would have on the target categorization response. Within the framework of symbolic knowledge theories, however, prime modality is not expected to an influential factor nor is prime modality expected to interact with any of the other variables.

In our previous study (Kim et al., 2014), we found evidence for cross-modal priming that supported the intermodal event file as described by Zmigrod et al. (2009) and the multisensory framework described by Chen and Spence (2011). However, pictures and environmental sounds were not found to be equally effective as primes, and an unexpected finding from that study was the fact that, when neutral picture primes were presented in advance of sound targets, RTs were longer relative to all other priming conditions. Because we used a repetition priming paradigm and measured priming by comparing the advantage provided by related primes to neutral primes, it was unclear exactly why the delayed response was occurring. In a series of studies, we eliminated processing time and target modality switching as possible explanations; but, there are a number of other explanations. For example, some researchers who study the integration of auditory and visual information find asymmetrical interference effects when bimodal information is semantically incongruent (Suied et al., 2009; Yuval-Greenberg and Deouell, 2009). Yuval-Greenberg and Deouell (2009) studied object identification in response to congruent and incongruent bimodal stimuli and they found cross-modal interference when attention was directed at either the picture or the sound targets; but the effect was greater when visual stimuli interfered with auditory target recognition than when the auditory stimuli interfered with the visual targets. When the quality of the visual stimulation was degraded, however, then the interference effects across modality were similar. It is possible that the delayed RT in our previous study reflected an inability to ignore irrelevant pictures when environmental sounds are processed or when the prime/target relationship is overwhelmingly congruent.

Schneider et al. (2008) also investigated cross-modal priming by varying the congruence of prime-target pairs, and they found shorter RTs when semantically congruent pairs were compared to incongruent pairs. However, their study differed from ours in several important respects. Although they used both picture and sound targets, they did so in separate experiments and the members of the prime-target pairs were the exact same stimulus as the target or the same item but presented in a different modality (congruent) or different items in either the same or different modality (incongruent). In our experiment, members of the prime-target pair were always physically different representations to minimize perceptual priming effects with the unimodal pairs, and to be sure that any difference between the unimodal and cross-modal pairs were primarily due to access to semantic memory. In addition, we compared four different types of prime-target pairs. For the congruent pairs, the prime and target stimuli were different exemplars of the same item (i.e., two physically different dogs) or two different items from the same conceptual category (i.e., dog and a cat), while the incongruent pairs were either a neutral prime (abstract picture or a pure tone) or an item drawn from a different response category from the target. Lastly, our study used a different categorization task than Schneider et al. (2008). They asked participants to indicate whether the target would fit in a shoebox—a size judgment task that may have required participants to visualize the target item or may have been easier with a picture rather than a sound representation as the target. With categorization of the targets as man-made or natural (Experiment 1) or animal or musical instrument (Experiment 2), there is no reason to suggest any advantage when either picture or environmental sound targets were presented.

By using unimodal and cross-modal prime-target pairs and varying the congruence and modality of the members of the pairs we can explore priming effects in greater depth than previous studies (Schneider et al., 2008; Kim et al., 2014). In half of the conditions, primes were unrelated (either neutral or members of different conceptual categories) to the target item. By manipulating the relevance of the prime so that it is from the same category as the target on 50% of the trials, we eliminated any effect due to the prevalence of the congruence of the prime.

In particular, we are interested in whether the priming effects obtained with same item exemplars extend to different items drawn from the same conceptual category as the target, and whether primes drawn from conceptual categories that differ from the target interfere with categorization decisions. If auditory and visual features are integrated into an event file as suggested by Zmigrod et al. (2009) or share access to the same semantic information (Chen and Spence, 2011) then we would expect that auditory and visual primes would have similar influences on target processes but there may be some variation in the effects due to differences in audio-visual processing which would underlie interaction effects or effects of target modality. For example, we would expect a larger priming effect with unimodal rather than cross-modal primes because of the format similarity in processing prime-target pairs. If, however, knowledge representation is symbolic and amodal (Pylyshyn, 1999; Riesenhuber and Poggio, 2000), then we would expect that congruent/incongruent primes would influence target RTs to a greater degree than target/prime modality. Target/prime format similarity would not matter because of the symbolic nature of the semantic representation. However, both theoretical frameworks would be similar in their prediction of a congruity effect and in their expectation for stronger congruity effects in Experiment 2 than Experiment 1 because the categorization response is at a lower level of the semantic network.

## MATERIALS AND METHODS

### PARTICIPANTS

The participants were 32 (29 female) undergraduate students from the University of North Carolina at Charlotte who were at least 18 years of age (*M*_age_ = 21, SD_age_ = 5.67), spoke English as their primary language, had normal hearing and vision (or corrected-to-normal vision) with no history of auditory or visual impairment. They participated to obtain extra credit points toward their psychology class grade. This study was approved by the Institutional Review Board at the University of North Carolina at Charlotte and informed consent was obtained prior to participation.

### MATERIALS

The stimulus set consisted of two picture and environmental sound exemplars of 80 category items that participants identified in the previous experiment (Kim et al., 2014) as belonging to the categories of man-made and natural things. A list of the items is presented in the Appendix and comparison data regarding picture and sound equivalence is available in Kim et al. (2014). The pictures and digitized sounds were selected from databases and clipart files to represent common and easily recognizable environmental sounds and pictures. There were an equal number of items from each of the categories—man-made and natural things.

Sound files were 32-bit stereo WAV files (sampling rate: 22,050 Hz) taken from Marcell et al. (2000) list of 120, and also from the internet (http://www.freesound.org). They were sounds taken from real world people, objects, and events as opposed to simulated sounds. The sounds were edited in Audacity 1.2.5 to a length of 750 ms for one exemplar of each item and 1 s for the other exemplar. Sound intensities were adjusted and delivered binaurally through headphones (Labtec Elite 825) at approximately 65 dB SPL.

Pictures were jpeg files resized using Adobe Photoshop to 4 cm × 4 cm. They were selected from clip art, the internet, and normed lists (Bonin et al., 2003; Rossion and Pourtois, 2004). As with the sounds, the pictures were programmed so that one exemplar of each item would be presented for 750 ms and the other for 1 s. There were five additional neutral pictures, which were taken from the pool of textured patterns available in Adobe Photoshop and five additional neutral tones generated by Audacity at each of the following frequencies—200, 400, 600, 800, and 1,000 Hz. The neutral stimuli were edited in the same way as the other stimulus items and, although they did not contain any object information, they were similar to the other pictures and environmental sounds in such physical characteristics as size, duration, loudness, and coloring.

Trials were blocked by target modality and the 80 category items within each block were randomly assigned to one of the eight-prime modality (picture or environmental sound) by prime type (same item, same category, neutral, or different category) conditions within each of the two target blocks. There were 10 items (five from each of the two categories – man-made and natural things) assigned to each of the 16 experimental conditions.

Four files were created in SuperLab 4.5 to counterbalance the items across the two target modality conditions, the two prime modalities, and the congruent and incongruent prime/target pairings. Each of the category items were used twice—once in each of the two target modalities except for the neutral stimuli, which were each used eight times as primes (four times in each of the target blocks of trials). Participants were randomly assigned to one of the four files. The stimuli were presented on an iMac computer with a 20^′′^ flat screen. SuperLab 4.5 was used to control stimulus presentation and data collection.

Congruent prime-target pairs were made up of items that were representations of the same item, or different items but members of the same conceptual category such as a representation of a dog and a cat. For the same item pairs, the cross-modal pairs consisted of a sound and picture generated from the same item, while the unimodal pairs had two physically different pictures or sounds generated by two exemplars of the same item (such as two different cats). For the same category condition, an effort was made to make sure that the primes were paired with target items from the same category level in the semantic network. The categories for man-made items were instruments, tools, and vehicles; and the natural item categories were animals, people, birds, insects, and natural events. For example, with an animal or a musical instrument as a target, then the prime would be another animal or musical instrument.

Incongruent pairs consisted of primes and targets that were from the two different target response categories (man-made vs. natural), while neutral pairs represented instances in which a target item was paired with a neutral prime that did not provide any information (abstract picture or a pure tone) that was related to the target item.

### PROCEDURE

**FIGURE 1:**
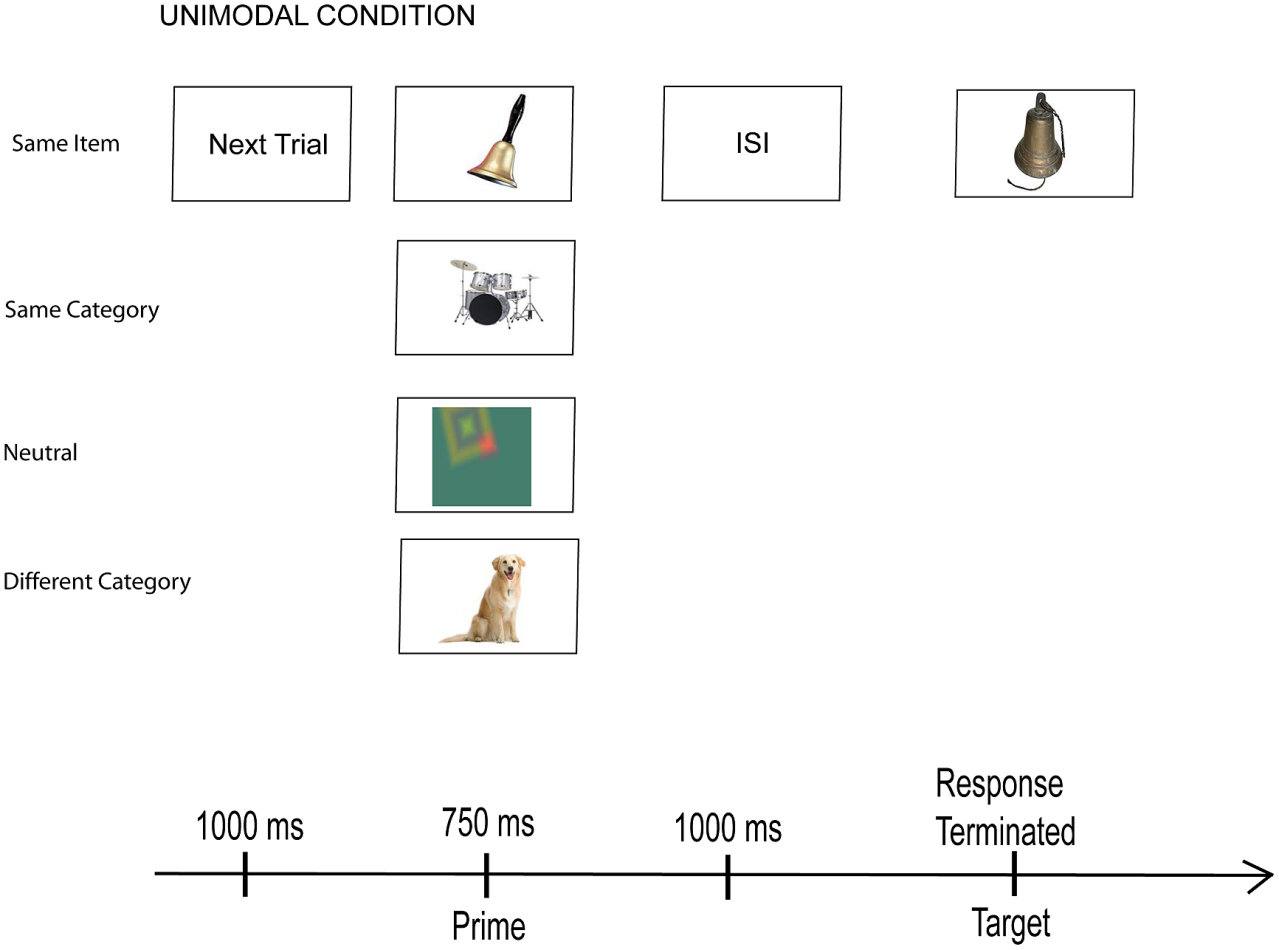
**Sequence of events on every trial.** An example of the four unimodal conditions with picture targets from Experiment 1.

**Figure 1** shows the events that occurred on each trial. A signal (next trial) indicated the beginning of each trial and provided a 1 s inter-trial interval. It was followed by the prime, which appeared for 750 ms. After a 1000 ms ISI (when a fixation cross appeared in the center of the screen), the target appeared. Participants were instructed to expect a pair of stimulus events on each trial, to look or listen to the first, and then to classify the second as belonging to the category of either man-made or natural things. They made a key press response to indicate their choice and end the trial.

Participants sat 30 cm from the computer screen in a well-lighted room, wore stereo headphones, and were run individually in 45 min sessions. They were instructed to expect two events on each trial and to respond to the second event by categorizing it as man-made or natural. In the instructions, man-made was defined as a picture or an environmental sound event that represents something people fabricate such as a car or a musical instrument, while natural was a sound or picture of a living thing or a natural resource or event such as water or lightning. They then practiced learning the response associations— a keystroke of “f” indicated a categorization of man-made while “j” indicated natural. A note positioned at the bottom of the monitor reminded the participants at all times about the response associations. In all, there were 160 experimental trials divided into two blocks of trials, one for each of the target modality conditions. Within each block there was a random arrangement of the 10 prime-target pairs in each of the eight experimental conditions. Participants also had 20 practice trials before the experimental session. Reaction times were measured from the onset of the target event until the participant’s key press and participant responses to each trial (RTs and key presses) were saved to an electronic file.

### RESULTS

Response times were trimmed if less than 200 ms or greater than 6000 ms. (One percent of responses were trimmed.) Additionally, the data from two of the participants were not included in the analysis because their performance was at chance for several of the experimental conditions. From the remaining 30 participants, mean correct RTs were computed across the 10 trials within each of the experimental conditions. The trimmed correct RTs and proportion of incorrect responses were analyzed with separate mixed ANOVAs to test for the between-groups effect of counterbalanced lists and the within effects of target modality, prime modality and prime type (same item, same category, neutral, and different category). A significance level of 0.05 was used for all statistical tests, and where appropriate the Greenhouse–Geisser correction was made to the *p*-value to protect against possible violations of the sphericity assumption.

#### Reaction times

**FIGURE 2:**
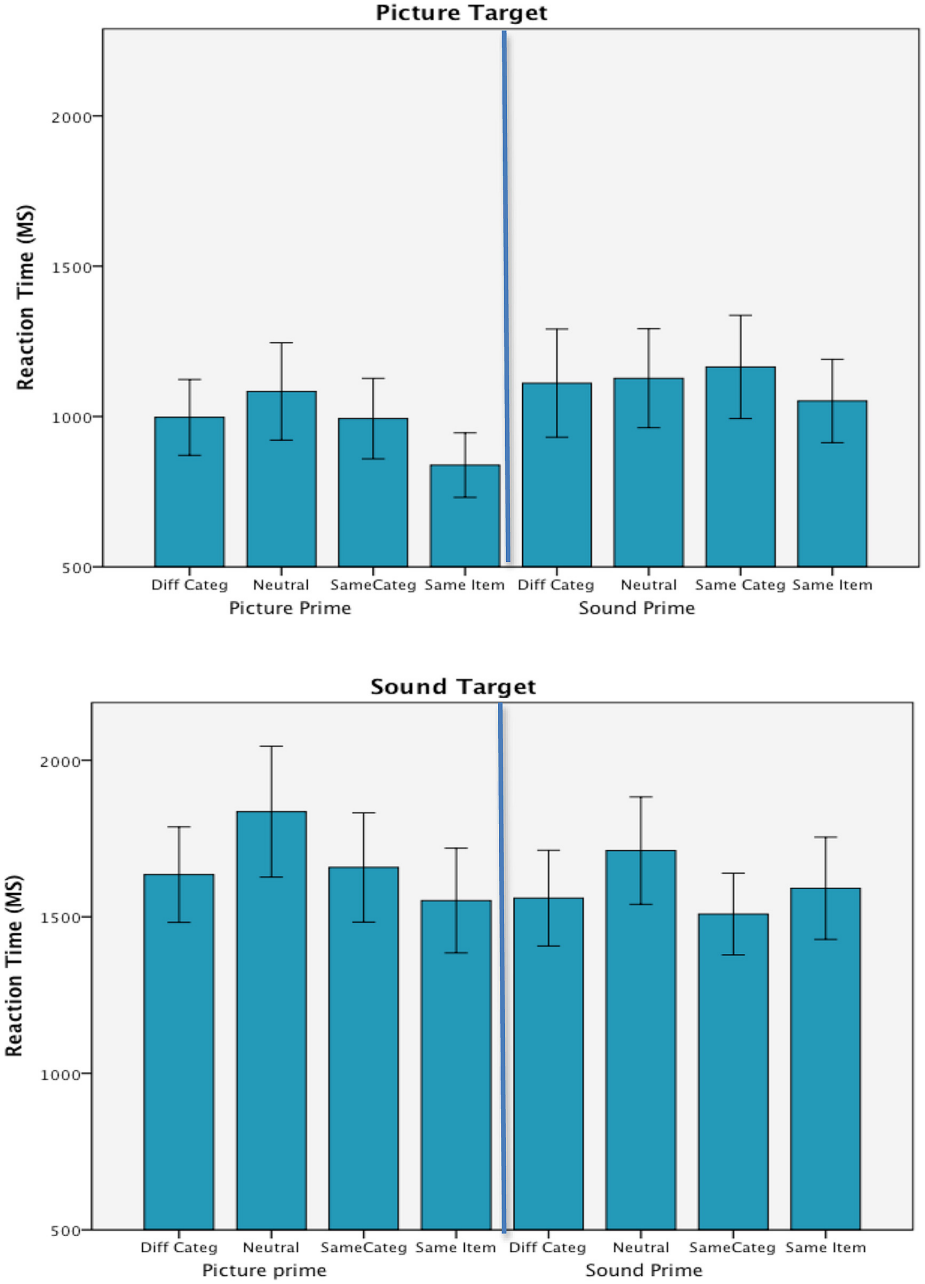
**Mean correct reaction times in each of the experimental conditions from Experiment 1.** Error bars are 95% confidence intervals.

**Figure 2** presents the mean trimmed correct RTs in each of the experimental conditions. The ANOVA did not show any between group effect of list, *F* < 1; and list did not interact with either target modality, *F*(3,26) = 1.96, *p* = 0.144, prime modality, *F* < 1, prime type, *F* < 1, or in any of the interactions with the experimental variables.

#### Modality effects

Consistent with the our previous work (Kim et al., 2014), however, responses to the environmental sound targets (*M* = 1631 ms) took longer than responses to picture targets (*M* = 1045 ms), *F*(1,26) = 166.81, *p* < 0.001, $\eta_{p}^{2}$ = 0.87; and there was a main effect of prime type, *F*(3,78) = 11.53, *p* < 0.001, $\eta_{p}^{2}$ = 0.31. Target modality was also found to interact with both prime type, *F*(3,78) = 4.17, *p* = 0.009, $\eta_{p}^{2}$ = 0.14 and prime modality, *F*(1,26) = 20.23, *p* < 0.001, $\eta_{p}^{2}$ = 0.44; and there was an additional interaction of prime type by prime modality, *F*(3,78) = 4.48, *p* < 0.006, $\eta_{p}^{2}$ = 0.15. The two-way interaction between target and prime modality resulted from the fact that RTs to targets were faster with unimodal rather than cross-modal primes. Picture targets were responded to more quickly when primed by pictures and similarly sound targets led to faster RTs when preceded by sound rather than picture primes. Because of their relevance to the congruency effect, the other interactions with prime type are discussed below. The three way interaction of target modality by prime modality by prime type was not significant, *F* < 1, and there was no main effect of prime modality, *F*(1,26) = 2.03, *p* = 0.166, $\eta_{p}^{2}$ = 0.07.

#### Priming effects

To measure the priming effect, which is the main focus of this study, follow-up repeated measures ANOVAs compared congruent/incongruent prime types within each of the target modalities. These results show a priming effect only when processing picture targets. Moreover, the effect was limited to same item primes and was not apparent when same category primes appeared in advance of the picture targets. A priming effect was not evident when categorizing sound targets. Instead, there was a delay in the RTs to the sound targets that resulted from the advanced presentation of neutral primes that were not related to the target categorization task. Under both target conditions, however, the presentation of primes from a different category than the target did not have an effect on target processing that was any different from same category primes.

Categorization times with picture targets (shown in the top panel of **Figure 2**) were faster when picture rather than sound primes were presented in advance, *F*(1,29) = 27.10, *p* < 0.001, $\eta_{p}^{2}$ = 0.48; and when same item primes were used in comparison to all other types of primes, *F*(3,87) = 8.41, *p* < 0.001, $\eta_{p}^{2}$ = 0.23. Pairwise *t*-tests (Bonferroni corrected) within the prime type main effect (*p*s < 0.01) showed that same item primes (944 ms) were significantly faster than all other prime types and that none of the other prime types differed from each other. Importantly, there were no differences between the neutral (1105 ms) and incongruent primes (1054 ms), and same category primes (1078 ms) did not provide any processing advantage when compared to neural or incongruent primes. The prime type by prime modality interaction failed to reach significance, *F*(3,87) = 2.79, *p* = 0.062, $\eta_{p}^{2}$ = 0.09.

The ANOVA on the categorization times in response to sound targets showed the same main effects as the previous analysis, but the pattern of findings differed. The means for these effects are shown in the bottom panel of **Figure 2** together with their respective 95% confidence intervals. When categorizing environmental sounds, sound primes lead to faster RTs than picture primes, *F*(1,29) = 5.21, *p* = 0.030, $\eta_{p}^{2}$ = 0.15; and the effect of prime type, *F*(3,87) = 9.57, *p* < 0.001, $\eta_{p}^{2}$ = 0.25, resulted from longer RTs to neutral primes (1774 ms) in comparison to all other prime types. Bonferroni corrected *t*-test comparisons showed that the advance presentation of same item primes (1571 ms) differed from neutral primes (*p* = 0.001) but not incongruent primes (1597 ms) and the only differences among the prime type conditions were the comparisons to the neutral primes. Also, prime type did not interact with prime modality, *F*(3,87) = 1.82, *p* = 0.164, $\eta_{p}^{2}$ = 0.06.

#### Errors

The average percent of errors ranged from 2 to 7% of the responses in each of the 16 experimental conditions and there was no evidence in the error data that participants traded speed for accuracy in any of the experimental conditions that were tested. There were more errors in response to environmental sound (5%) than picture targets (3%), *F*(1,26) = 5.01, *p* = 0.034, $\eta_{p}^{2}$ = 0.16; and when sounds (5%) rather than pictures (4%) were used as primes, *F*(1,26) = 5.78, *p* = 0.024, $\eta_{p}^{2}$ = 0.18. Moreover, there was a two-way interaction of target and prime modality, *F*(1,26) = 11.75, *p* = 0.002, $\eta_{p}^{2}$ = 0.311, that was due to a slight elevation in the error rate when unimodal sound pairs (6%) were compared to picture (3%) or cross-modal pairs (3%). The error rate did not vary as a function of prime type, *F* < 1, but prime type interacted with prime modality, *F*(3,78) = 3.71, *p* = 0.015, $\eta_{p}^{2}$ = 0.13. The interaction resulted from a low error rate (2%) when same item or same category picture primes were compared to the other conditions within this interaction. There was also a three-way interaction of target modality by prime modality by prime type, *F*(3,78) = 5.35, *p* = 0.002, $\eta_{p}^{2}$ = 0.17. However, the error rates in these 16 conditions only varied slightly, and it is unclear what caused the higher order interaction effect.

There was also a difference in the error rate as a function of the counterbalanced lists, *F*(3,26) = 3.31, *p* = 0.036, **$\eta_{p}^{2}$** = 0.28, where one list with a 7% error rate was found to be different from another with only a 2% error rate. This variable was also found to interact in two three-way interactions [list by prime modality by prime type *F*(9,78) = 2.14, *p* = 0.036, **$\eta_{p}^{2}$** = 0.20, and list by target modality and prime modality, *F*(3,26) = 5.20, *p* = 0.006, **$\eta_{p}^{2}$** = 0.38], and in a 4-way interaction with all of the other experimental variables, *F*(9,78) = 2.73, *p* = 0.008, **$\eta_{p}^{2}$** = 0.24. The complex effects with counterbalanced lists resulted from elevated error rates, which ranged from 11 to 18%, in response to incongruent and neutral primes for two of the lists. The elevated error rates were traced to the data from two participants assigned to different list conditions who had high error rates relative to all other participants in neutral and incongruent prime conditions. There were six instances of error rates that exceeded 10%.

### DISCUSSION

The findings of this study are notable in showing a strong effect of target modality and two distinct patterns of conceptual priming effects within each. When processing picture targets and making a decision about membership in either a man-made or natural category, priming effects are found when either pictures or sounds derived from the same item as the target are presented in advance. However, the priming advantage does not extend to primes that represent other items in the same conceptual category as the target item.

When processing environmental sounds, there was a priming advantage only when the advanced presentation of either pictures or environmental sounds derived from the same item as the target were compared to neutral primes. Unexpectedly, no priming advantage was found when same item primes were compared to primes drawn from a different response category than the target. The data show clearly that the RT advantage when same item primes are compared to neutral primes result from a delayed response to the neutral primes rather than a priming advantage. By manipulating four different types of prime/target pairs, rather than the two used in our previous study (Kim et al., 2014), we could provide a comprehensive context for investigating the conceptual priming effects of environmental sounds and pictures, and as a result the evidence for a priming effect with environmental sound targets is much less evident.

The most puzzling aspect of these findings is the fact that primes drawn from same and different response categories did not differ in their influence on target processing. Same category primes did not show the same priming advantage as the primes that represented different exemplars of the same item and primes drawn from an incongruent response category did not interfere with target processing beyond the effect shown by neutral primes. This result is at odds with Schneider et al. (2008), however, in their work they only compared two types of primes (congruent and incongruent) and they used the same physical item for the congruent primes, which suggests perhaps an additional advantage of perceptual priming. Since we used different exemplars of the same object, our results reflected primarily conceptual priming. We do acknowledge, however, that, although sound or picture primes derived from another exemplar of the same item as the target are not the same physically, they may in some cases share more similar physical features than two different items from the same category. So, some limited perceptual priming may also be involved in our priming results.

Another likely explanation may be related to the use of response categories that were at a higher level in the semantic network than the lower level categories used for prime/target category pairings (Rosch et al., 1976), or perhaps there were variations in semantic level among the categories used for the pairings. Either of these may have added noise to the data and masked the priming advantage of congruent over incongruent pairs. If the target response used a basic level concept rather than a super-concept then the findings may have been different. Experiment 2 was developed to address that issue by using basic or lower level conceptual categories and testing for unimodal and cross-modal priming.

## EXPERIMENT 2

As in the previous experiment, we tested for conceptual priming with prime-target pairs that vary in congruence and modality, but for this experiment the conceptual task involved category discriminations at a lower level (animals or musical instruments) in the semantic network than the superordinate level (natural or man-made) tested previously. Asking participants to decide whether objects belong to a category of musical instruments or animals is an intermediate level that is more closely associated with the basic semantic level than the task used in Experiment 1. Decisions regarding the basic level have been found to be those most frequently encountered, and to be the most informative when discriminating among categories (Rosch et al., 1976; Jolicoeur et al., 1984). Also, by using intermediate level category pairing rather than the superordinate, we reduced the variations in semantic level between the prime-target pairs. For these reasons, we expected that animal/musical instrument decisions would be a more sensitive test of the congruency effect than the natural versus man-made decisions used in the previous experiment.

### METHOD

The participants were 28 (21 female) undergraduate students drawn from the same participant pool as the previous experiment who were at least 18 years of age (*M*_age_ = 20.67, SD_age_ = 2.67), had normal hearing and vision ( or corrected to normal) with no history of auditory or visual impairment. They participated to obtain extra credit points toward their psychology class grade. Sixty-eight percent of the sample was white, 14% African American and 18% other. None of the participants were in the previous experiment. This study was approved by the Institutional Review Board at the University of North Carolina at Charlotte, and informed consent was obtained prior to participation.

The stimulus set was a subset of the items used in Experiment 1 with the addition of the following items; double bass, flute, and clarinet. There were two picture and environmental sound exemplars for each of the 40 items that were equally divided between animals and musical instruments. Each item was used twice as a target (once as a picture and once as an environmental sound) and twice as a prime (once as a picture and once as an environmental sound).

Trials were blocked by target modality and the 40 category items within each block were randomly assigned to one of the eight prime modality by prime congruency (same, different category as the target) conditions. There were 10 items (five from each of the two response categories) assigned to each of the eight experimental conditions. Four files were created to counterbalance the items across the two target modalities, two prime modalities, and two congruency conditions.

Participants sat 30 cm from the computer screen in a well-lighted room, wore stereo headphones, and were run individually in 30 minute sessions. After reading the instructions and learning the response associations, they participated in 20 practice trials. The experimental session consisted of 80 trials separated into two blocks of 80. In all other respects, the procedure for this experiment was the same as the previous experiment.

### RESULTS

Response times were trimmed if less than 200 ms or greater than 4500 ms. (Less than 1% of the responses were trimmed.) The trimmed correct RTs and proportion of incorrect responses were analyzed with separate mixed ANOVAs to test for the between-group effect of counterbalanced lists and the within effects of target modality, prime modality, and prime/target congruence. A significance level of 0.05 was used for all statistical tests.

#### Reaction times

**FIGURE 3:**
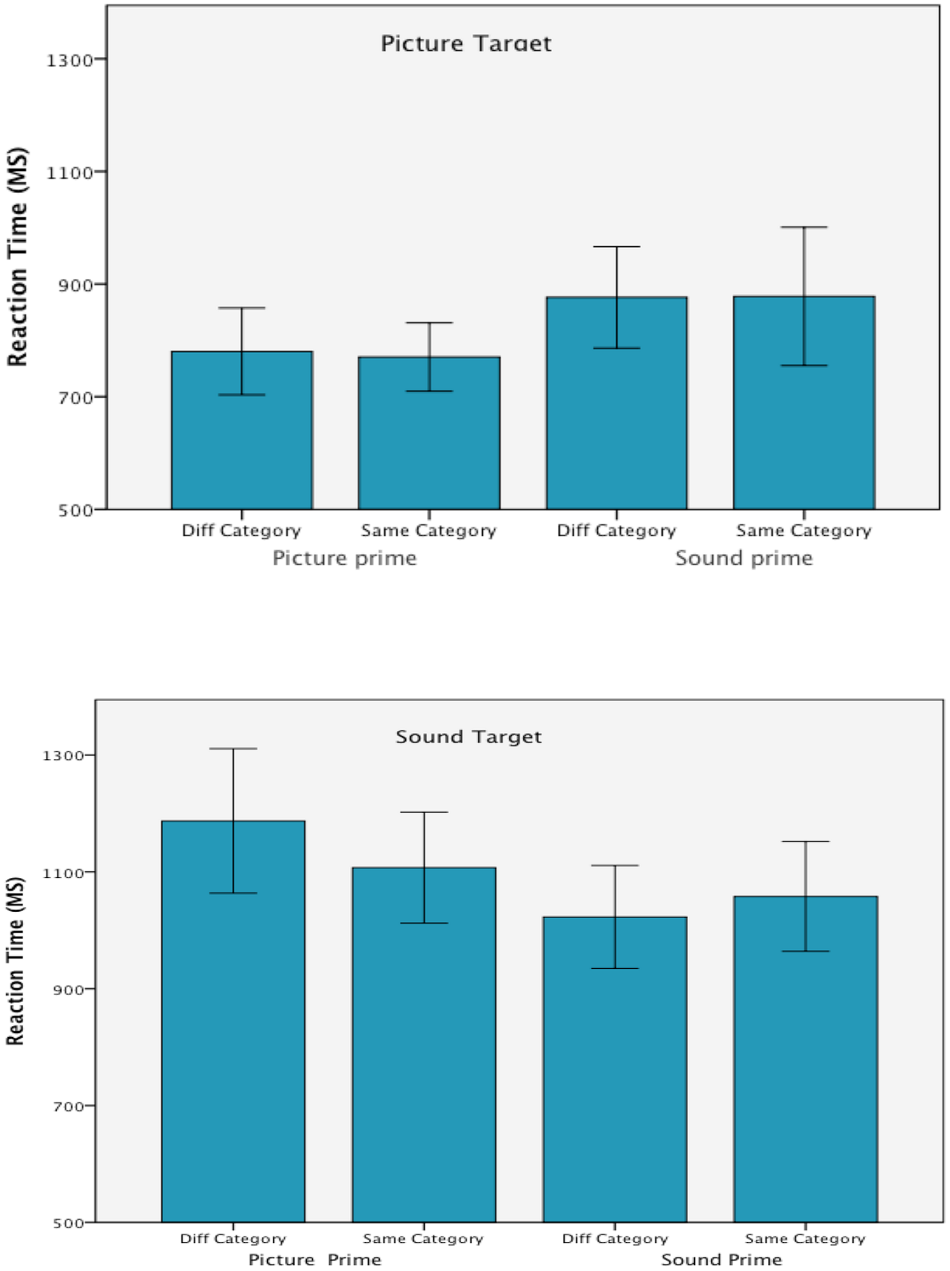
**Mean correct reaction time in each of the experimental conditions from Experiment 2.** Error bars are 95% confidence intervals.

**Figure 3** presents the mean trimmed correct RTs in each of the experimental conditions. The ANOVA did not show any between group effect of list, *F*(3,24) = 2.20, *p* = 0.114, $\eta_{p}^{2}$ = 0.22; and list did not interact with target modality, prime modality, prime/target congruence, *F*s < 1, or in any higher order interactions.

#### Modality effects

As expected responses to environmental sound targets took longer (*M* = 1093 ms) than responses to picture targets, (*M* = 826 ms), *F*(1,24) = 66.30, *p* < 0.001, $\eta_{p}^{2}$ = 0.73 and there was an interaction between target and prime modality, *F*(1,24) = 16.28, *p* < 0.001, $\eta_{p}^{2}$ = 0.40 that was consistent with the previous experiment. Target responses were faster when preceded by unimodal rather than cross-modal primes. There was no main effect of prime modality, *F* < 1.

Overall RTs were not influenced by prime/target congruence, *F* < 1 and this variables did not interact with target modality, *F* < 1, prime modality, *F*(1,24) = 2.04, *p* = 0.167, $\eta_{p}^{2}$ = 0.08, or in a three way interaction with target and prime modality, *F*(1,24) = 2.68, *p* = 0.115, $\eta_{p}^{2}$ = 0.10.

#### Priming effect

Preceding the targets with same or different category primes made a difference only when environmental sound targets were preceded by cross-modal primes. The RTs analysis did not show an effect of congruency when picture targets were processed. These effects were confirmed by the follow-up ANOVAs that were conducted within each of the modality conditions.

The analysis on the picture targets showed an effect of prime modality, *F*(1,27) = 10.72, *p* = 0.003, $\eta_{p}^{2}$ = 0.28, but no effect of congruency and no interaction of prime modality by congruency, *F*s < 1. When processing sound targets, however, the target/prime congruency was found to interact with prime modality, *F*(1,27) = 4.08, *p* = 0.053, $\eta_{p}^{2}$ = 0.13. The interaction resulted from a processing advantage when congruent rather than incongruent category primes preceded the sound target but only with picture primes, *t*(26) = 1.72, *p* = 0.048. When sound primes were presented in advance of the sound targets there was no effect of congruency, *t* < 1. There was no main effect of congruency, *F* < 1, but RTs were faster when sound rather than picture primes were used, *F*(1,27) = 8.61, *p* = 0.007, $\eta_{p}^{2}$ = 0.24.

#### Errors

Across all of the experimental conditions, the error rate was 5% or less, and the analysis of the error data showed that none of the manipulated variables had a significant influence. Picture targets were categorized incorrectly on only 1.8% of the trials versus 3.3% for the environmental sound targets. There was no main effect of target modality, *F*(1,24) = 3.44, *p* = 0.076, $\eta_{p}^{2}$ = 0.13, prime modality or prime/target congruence, *F*s < 1; and these variables were not found to interact. The list effects that were found in Experiment 1 were not evident. There was no between group effect of list, *F* < 1, and list did not interact with any of the other variables. Since most of the list effects in Experiment 1 were attributed to the neutral prime type, and that condition was not included in this study, it is not surprising that list effects were absent.

### DISCUSSION

When the conceptual priming task involved discriminations at an intermediate level of the semantic network, primes from the same category facilitated target responses in comparison to different category primes but the effect was limited to environmental sound targets and cross-modal primes. An effect of prime/target congruency was not obtained when picture targets were processed or when sound targets were preceded by unimodal primes. This finding is consistent with the research of others who find asymmetrical interference effects across modalities (Yuval-Greenberg and Deouell, 2009). When processing sound targets it is more difficult to ignore incongruent pictures than sounds when they appear in advance of the target. However, with picture targets the congruency effect is negligible whether unimodal or cross-modal primes appear.

As expected, animal/musical instrument discriminations resulted in much shorter RTs on average (approximately 200 ms difference in picture discriminations and 500 ms difference in environmental sounds) when compared to superordinate discriminations. Otherwise, the findings were consistent with Experiment 1. There were large effects of target modality, and target and prime modality interacted showing that unimodal primes shortened RTs in comparison to cross-modal primes.

## GENERAL DISCUSSION

Examining conceptual priming in a comprehensive manner, we found two distinct patterns of priming effects using picture and environmental sound targets. With superordinate categorization responses, priming was obtained only with picture targets and only when same item primes rather than same category primes were presented. Processing environmental sound targets were delayed by neutral primes but not by different category primes. With categorization responses at an intermediate level in the semantic network, however, processing of environmental sounds targets were delayed by incongruent relative to congruent category primes but only if picture primes were presented. A congruence effect was not found with sound targets when sound primes appeared or in any of the conditions with picture targets.

When these findings are considered in light of the theoretical frameworks presented in the introduction, there is clear evidence in support of sensory integration either in the form of an intermodal event file (Zmigrod et al., 2009) or a multisensory framework (Chen and Spence, 2011). In both experiments prime modality interacted with target modality and RTs to targets were faster with unimodal rather than cross-modal primes. If knowledge is stored in an amodal representation, and there is no direct connection between audio-visual features of an item (Pylyshyn, 1999; Riesenhuber and Poggio, 2000), then prime modality would not be expected to modulate the effect of target modality. Although the strong effect of target modality is consistent with both theoretical frameworks, because it is based on format differences in processing picture and sound targets, the interaction effects between the prime and target modality are not.

Also, congruity effects were much more elusive than modality effects in both experiments. In Experiment 1 when superordinate categorization responses were measured there was a priming effect that was limited to only picture targets and constrained further to only picture and sound primes derived from the same item as the picture target. In Experiment 2, however, when the category task was closer to the basic level in the semantic network, then sound targets were found to be delayed by picture primes derived from different rather than the same conceptual categories from the target item. Taken together the lack of a difference between congruent/incongruent primes, and the finding of similar unimodal and cross-modal priming effects provides some evidence that auditory and visual features about a single item in a conceptual category may be more tightly connected than two different items from the same conceptual category. This finding is consistent with sensory integration in the intermodal event file described by Zmigrod et al. (2009).

The advanced presentation of environmental sounds derived from the same items as picture targets facilitated target categorization in the same way as picture primes. Similarly, when processing sound targets, picture, and sound primes yielded similar effects. The only exception was when picture primes interfered with environmental sound targets in Experiment 2. As indicated previously, these findings are consistent with previous research (Suied et al., 2009; Yuval-Greenberg and Deouell, 2009) and suggest that the increase in the RTs to environmental sound targets when paired with neutral pictures may have resulted from an inability to ignore the picture when processing sound targets.

Also, the delayed response to the sound targets when presented after the unrelated primes most likely resulted from the fact that the neutral primes were vague and not as identifiable as the other primes. The increase in error rate associated with this condition in Experiment 1 supports this interpretation. In effect, the presence of the abstract pictures and the pure tones in advance of the sound targets distracted attention from target processing. Interestingly, the delayed RT effect did not occur when neutral primes preceded picture targets, perhaps suggesting that visual processing is not as susceptible to attentional distractors, regardless of their modality.

The findings extend our previous work (Kim et al., 2014) with unimodal and cross-modal priming by manipulating prime/target congruency and by investigating conceptual priming at both a superordinate and intermediate level of the semantic network. Although the findings from Experiment 1 replicate the previous effort by showing both unimodal and cross-modal priming effects when same item primes are compared to neutral primes, it is clear that the previous explanation of the findings must be reconsidered.

These findings are limited by the nature of the stimulus items that are used—commonly recognized nouns and environmental sounds, and by the fact that only college students were tested. Whether they are generalizable to broader categories of participants and stimulus materials can only be determined by more research. Also, it is important to acknowledge that the use of two exemplars to represent the items listed in the appendix was done to minimize effects of perceptual priming in Experiment 1. But, it is entirely possible that the findings contained some influence of perceptual priming. There is no doubt that two exemplars of an item such as an animal or a musical instrument share more similar physical features that two different items for the same conceptual category and the greater physical similarity may have contributed to the unimodal priming effects obtained in Experiment 1. However, the fact that cross-modal primes were found to have similar influences on target categorization as the unimodal primes indicates that conceptual priming was also involved.

## Conflict of Interest Statement

The authors declare that the research was conducted in the absence of any commercial or financial relationships that could be construed as a potential conflict of interest.

## SUPPLEMENTARY MATERIAL

The Supplementary Material for this article can be found online at:

## References

[B1] Bonin P. Peereman R. Malardier N. Meot A. Chalard M. (2003). A new set of 299 pictures for psycholinguistic studies: French norms for name agreement, image agreement, conceptual familiarity, visual complexity, image variability, age of acquisition, and naming latencies. *Behav. Res. Methods Instrum. Comput.* 35 158–167 10.3758/BF03195507 12723790

[B2] Chen Y.-C. Spence C. (2011). Cross-modal semantic priming by naturalistic sounds and spoken words enhances visual sensitivity. *J. Exp. Psychol. Hum. Percept. Perform.* 37 1554–1568 10.1037/a0024329 21688942

[B3] Glaser W. R. Glaser M. O. (1989). Context effects in stroop-like word and picture processing. *J. Exp. Psychol.* 118 13–42 10.1037/0096-3445.118.1.13 2522504

[B4] Hommel B. (1998). Event files: evidence for automatic integration of stimulus-response episodes. *Vis. Cogn.* 5 183–216 10.1080/713756773

[B5] Hommel B. (2004). Event files: feature binding in and across perception and action. *Trends Cogn. Sci. (Regul. Ed.)* 8 494–500 10.1016/j.tics.2004.08.007 15491903

[B6] Hommel B. (2005). How much attention does an event file need? *J. Exp. Psychol. Hum. Percept. Perform.* 31 1067–1082 10.1037/0096-1523.31.5.1067 16262499

[B7] Jolicoeur P. Gluck M. A. Kosslyn S. M. (1984). Pictures and names: making the connection. *Cogn. Psychol.* 16 243–275 10.1016/0010-0285(84)90009-4 6734136

[B8] Kahneman D. Treisman A. Gibbs B. J. (1992). The reviewing of object files: object-specific integration of information. *Cogn. Psychol.* 24 175–219 10.1016/0010-0285(92)90007-O 1582172

[B9] Kim Y. Porter A. M. Goolkasian P. (2014). Conceptual priming with pictures and environmental sounds. *Acta Psychol. (Amst.)* 146 73–83 10.1016/j.actpsy.2013.12.006 24412837

[B10] Marcell M. M. Borella D. Greene M. Kerr E. Rogers S. (2000). Confrontation naming of environmental sounds. *J. Clin. Exp. Neuropsychol.* 22 830–864 10.1076/jcen.22.6.830.949 11320440

[B11] Pylyshyn A. (1999). Is vision continuous with cognition? A case for cognitive impenetrability of visual perception. *Behav. Brain Sci.* 22 341–423 10.1017/S0140525X99002022 11301517

[B12] Riesenhuber M. Poggio T. (2000). Models of object recognition. *Nat. Neurosci.* 3 1199–1204 10.10.38/81479 11127838

[B13] Rosch E. Mervis C. V. Gray W. D. Johnson D. M. Boyes-Braem P. (1976). Basic objects in natural categories. *Cogn. Psychol.* 8 382–439 10.1016/0010-0285(76)90013-X

[B14] Rossion B. Pourtois G. (2004). Revisiting Snodgrass and Vanderwart’s object pictorial set: the role of surface detail in basic-level object recognition. *Perception* 33 217–236 10.1068/p5117 15109163

[B15] Schneider T. R. Engel A. K. Debener S. (2008). Multisensory identification of natural objects in a two-way cross-modal priming paradigm. *Exp. Psychol.* 55 121–132 10.1027/1618-3169.55.2.121 18444522

[B16] Suied C. Bonneel N. Viaud-Deouell L. (2009). Integration of auditory and visual information in the recognition of realistic objects. *Exp. Brain Res.* 194 91–102 10.1007/s00221-008-1672-6 19093105

[B17] Yuval-Greenberg S. Deouell L. (2009). The dog’s meow: asymmetrical interaction in cross-modal object recognition. *Exp. Brain Res.* 193 603–614 10.1007/s00221-008-1664-6 19066869

[B18] Zmigrod S. Spapé M. Hommel B. (2009). Intermodal event files: integrating features across vision, audition, taction, and action. *Psychol. Res.* 73 674–684 10.1007/s00426-008-0163-5 18836741 PMC2708333

