# Supplementary material for: Target categorization with primes that vary in both congruency and sense modality
Source: Front Psychol. 2015 Jan 23;6:20. doi: 10.3389/fpsyg.2015.00020 (PMC4304167; doi:10.3389/fpsyg.2015.00020)
Supplement: Supplementary file 1 [file Data_Sheet_1_.DOCX]

| **Man-Made** | **Natural** |
| --- | --- |
| Accordion | Baby |
| Bagpipe | Baby chicks |
| Banjo | Bald eagle |
| Basketball | Bee |
| Bell | Bird |
| Boat | Cat |
| Bongos | Chicken |
| Bowling pins | Cow |
| Camera | Cricket |
| Car | Crow |
| Cash register | Dog |
| Champagne bottle | Dolphin |
| Coins | Donkey |
| Cymbal | Drop of water |
| Doorbell | Duck |
| Drum | Fly |
| Elevator | Frog |
| Fireworks | Goat |
| Glass breaking | Goose |
| Gong | Gorilla |
| Guitar | Hawk |
| Hammer | Heartbeat |
| Harmonica | Horse |
| Harp | Laughing |
| Helicopter | Leopard |
| Lawn mower | Lion |
| Machine gun | Monkey |
| Motorcycle | Mosquito |
| Organ | Ocean |
| Piano | Owl |
| Police siren | River |
| Saxophone | Rooster |
| Soda can | Seagull |
| Telephone | Seal |
| Toilet | Sheep |
| Train | Snoring |
| Trumpet | Thunder |
| Violin | Tiger |
| Wind chime | Turkey |
| Zipper | Wolf |

Appendix. A list of the stimulus items used in each of the target categories
